# Effect of Maternal Obesity on Fetal Interventricular Septum Volume: A Three-Dimensional Ultrasound STIC and VOCAL Study

**DOI:** 10.3390/jcm15145555

**Published:** 2026-07-15

**Authors:** Stefano Raffaele Giannubilo, Camilla Grelloni, Elisa Carboni, Dayana Quintili, Mariarosaria Motta, Dario Colacurci, Chiara Murolo, Alessandro Cecchi, Giuseppe Maria Maruotti, Andrea Ciavattini

**Affiliations:** 1 Department of Clinical Sciences, Polytechnic University of Marche, 60123 Ancona, Italy; c.grelloni@pm.univpm.it (C.G.);; 2 Centro Unico Regionale SODS, Diagnosi Prenatale di II Livello, 60025 Loreto, Italy; 3 Department of Public Health, University of Naples Federico II, 80131 Naples, Italydario.colacurci@unina.it (D.C.);

**Keywords:** obesity, interventricular septum volume, STIC-VOCAL, 3D-echocardiography

## Abstract

**Objective**: To assess correlations between maternal obesity and fetal interventricular septum (IVS) volume measured by means of spatiotemporal image correlation and Virtual Organ Computer-Aided Analysis (STIC-VOCAL), after adjustment for gestational age. **Methods**: This cross-sectional observational study included 88 consecutive singleton pregnancies undergoing routine obstetric ultrasound at our center between January 2024 and December 2025. Fetal IVS volume was assessed using STIC-VOCAL, and its association with maternal characteristics was evaluated by means of univariate and multivariable regression analyses adjusted for gestational age. **Results**: Fetal IVS volume increased with estimated fetal weight (r = 0.860, *p* < 0.0001) and gestational age (r = 0.891, *p* < 0.0001), which represented the main determinant of absolute fetal cardiac volume. The association between maternal obesity and fetal IVS volume was not evident on univariate analysis (U = 987.5, *p* = 0.874) but became significant after adjustment for gestational age in multivariable regression models (β = +89.1 mm^3^, *p* < 0.0001). At comparable gestational ages, maternal obesity was associated with an adjusted increase in fetal IVS volume of approximately 22.6 mm^3^. **Conclusions**: Maternal obesity was independently associated with a modest increase in fetal IVS volume after adjustment for gestational age. STIC-VOCAL volumetry may provide a sensitive tool for detecting subtle fetal cardiac structural changes, although these findings remain exploratory.

## 1. Introduction

The global burden of obesity has reached epidemic proportions, with nearly 1.9 billion adults worldwide living with this condition [1]. This trend is particularly alarming in the obstetric population, as in some regions, more than half of women enter pregnancy with overweight or obesity [2]. Beyond the immediate risks to maternal health, such as preeclampsia and gestational diabetes mellitus (GDM), maternal obesity has important implications for the long-term health of the offspring.

Excess adiposity is characterized by a chronic low-grade inflammatory state and profound metabolic disturbances, including insulin resistance and elevated circulating levels of leptin and pro-inflammatory cytokines [3]. During gestation, this altered maternal metabolic milieu may influence fetal developmental programming through exposure to a complex and suboptimal intrauterine environment [4]. Specifically, these metabolic disturbances are associated with oxidative stress and mitochondrial dysfunction, which may adversely affect the fetal heart during the critical period between the third and eighth weeks of gestation, when it is particularly vulnerable to environmental, genetic, and metabolic insults [5,6,7,8,9]. Moreover, insulin acts as a potent cardiomyotrophic hormone, and fetal hyperinsulinemia may directly stimulate myocardial hyperplasia and hypertrophy [10]. The association between maternal obesity and congenital heart defects (CHD) has been consistently demonstrated in several meta-analyses and prospective studies. The risk of CHD progressively increases with maternal BMI, with obese women (BMI ≥ 30 kg/m^2^) showing a significantly higher likelihood of offspring affected by interventricular septal defects, atrial septal defects, coarctation of the aorta, and great vessel anomalies [11,12]. Beyond structural congenital abnormalities, growing evidence suggests that the intrauterine environment associated with maternal obesity may also promote adverse fetal cardiac remodeling, with potential long-term cardiovascular consequences persisting from fetal life into adulthood [4]. Among the various cardiac structures involved, the interventricular septum (IVS) has emerged as a particularly sensitive marker of obesity-related fetal cardiac remodeling [2,13]. Recent clinical studies have consistently demonstrated that fetuses of obese women exhibit greater IVS thickness compared to those of lean mothers, with these changes detectable as early as the first or second trimester [14]. Importantly, this septal thickening does not appear to be merely a transient finding; rather, it represents an early localized manifestation of myocardial hypertrophy that has been shown to persist into early childhood and to be associated with an increased risk of hypertension and heart failure later in life [4]. Historically, the assessment of fetal myocardial thickness has relied on conventional M-mode and two-dimensional (2D) ultrasound. However, 2D techniques may have limited sensitivity, particularly in cases of mild hypertrophy or when maternal abdominal subcutaneous adipose tissue compromises image quality. To overcome these limitations, advanced imaging approaches based on four-dimensional (4D) ultrasound, including Spatiotemporal Image Correlation (STIC) and Virtual Organ Computer-Aided Analysis (VOCAL), have been introduced [10]. These technologies provide superior delineation of ventricular wall borders, enabling highly accurate volumetric assessment of the fetal interventricular septum. By moving beyond conventional linear thickness measurements toward 3D/4D volumetric assessment, clinicians may achieve earlier and more sensitive detection of fetal cardiac hypertrophy. Despite the well-established association between maternal BMI and fetal cardiac remodeling [2,13,15,16], data specifically addressing the relationship between maternal obesity and fetal IVS volume throughout gestation remain limited. This gap is clinically relevant, as the IVS plays a critical role in cardiac function, and changes in its volume may reflect alterations in ventricular geometry driven by fetal hemodynamic adaptation. A better understanding of these relationships may help clarify the structural correlates of maternal obesity in fetal life. Therefore, the aim of this study was to evaluate the association between fetal IVS volume and maternal characteristics, while adjusting for gestational age.

## 2. Materials and Methods

We conducted a cross-sectional observational study involving pregnant women referred to the Regional Center for Prenatal Diagnosis (Loreto, Marche, Italy), in collaboration with the Clinic of Obstetrics and Gynecology of the Polytechnic University of Marche (Ancona, Italy), between January 2024 and December 2025. All participants underwent fetal ultrasound between 18 + 0 and 36 + 6 weeks of gestation. Examinations were performed according to the standard protocols adopted at our center and in accordance with the guidelines of the International Society of Ultrasound in Obstetrics and Gynecology (ISUOG) [17]. During each examination, volumetric assessment of the fetal heart was performed using the STIC-VOCAL technique. Eligible participants included singleton pregnancies with availability of a diagnostic-quality STIC clip and complete maternal clinical data, including age, pre-pregnancy BMI, current BMI, parity, and cardiovascular risk factors. Pregnancies complicated by fetal cardiac anomalies or maternal and obstetric conditions potentially affecting fetal growth or cardiac development—including hypertension, diabetes mellitus, fetal growth restriction, metabolic or endocrine disorders, placental abnormalities, chromosomal abnormalities, and fetal malformations—were excluded. The following maternal variables were collected for each participant: maternal age (years), pre-pregnancy body mass index (BMI; calculated as weight in kilograms divided by height in meters squared), current BMI, gestational BMI increase, and parity (defined as the number of previous deliveries). Pregnancy-related variables were also recorded, including gestational age (weeks + days), fetal sex and estimated fetal weight (grams), calculated using conventional fetal biometry (biparietal diameter, head circumference, femur length, and abdominal circumference). Maternal obesity was defined as a BMI ≥ 30 kg/m^2^ at the time of ultrasound examination, according to World Health Organization (WHO) criteria [18].

### 2.1. Cardiac Volume Acquisition (STIC) and Volumetric Analysis (VOCAL Method)

All participants underwent fetal ultrasound examination between 18 + 0 and 36 + 6 weeks of gestation using a Voluson™ Expert 22 ultrasound system (GE Healthcare Ultrasound, Zipf, Austria) equipped with an eM6C G3 electronic matrix 4D convex volume transducer (2.0–7.0 MHz) and integrated STIC and VOCAL software packages (BT22; 2022). All sonographic examinations were performed by experienced operators with more than 5 years of expertise in fetal cardiac 3D ultrasonography. Following an initial two-dimensional (2D) ultrasound examination for fetal biometry assessment and fetal sex determination, four-dimensional (4D) STIC was used for fetal cardiac volumetric analysis.

The acquisition protocol followed a standardized approach:

- Preparation: First, a systematic 2D evaluation of the fetal heart was performed to obtain a standard four-chamber view
- 4D-STIC time: As previously described [19], 4D-STIC volumes were acquired during periods of fetal quiescence and maternal breath-holding to minimize motion artifacts. Image acquisition was initiated from a transverse four-chamber view, while avoiding inclusion of the fetal spine to reduce posterior acoustic attenuation. The acquisition angle was set between 20 and 40°, and acquisition time ranged from 10 to 15 s to ensure inclusion of the entire fetal heart and great vessels, including the four-chamber and outflow tract views.
- Selection: For each fetus, at least three volumetric datasets were acquired, and the dataset with the best image quality and lowest degree of motion artifacts.

**Figure 1:**
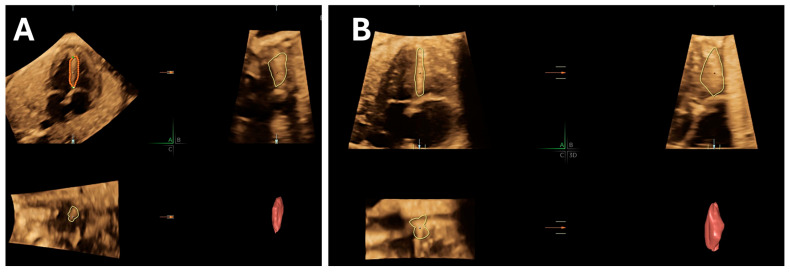
Representation of STIC acquisition and VOCAL Analysis in two fetuses, respectively at 26 + 1 (**A**) and 34 + 0 (**B**) gestational weeks.

STIC clips from multiple cardiac cycles were processed offline using VOCAL software for manual volumetric quantification of the IVS. The VOCAL procedure was standardized as follows:

- Orientation: The selected volume was displayed in multiplanar mode and rotated around the horizontal axis to position the apex of the fetal heart at the “12 o’clock” position.
- Cardiac Cycle Synchronization: All measurements were taken during the initial diastolic phase of the cardiac cycle, when the ventricles are at their widest and the septum is least contracted.
- Manual Tracing: Using the axial plane as a reference, the operator manually delineated the perimeter of the interventricular septum, tracking the endocardial edges between the left and right ventricles.
- Sequential Rotation: The software was set for a sequential rotation of 30 degrees. This manual tracing process was repeated six times to cover the entire volume of the structure.
- Volume Calculation: Upon completion of the full rotation, the software automatically calculated the total IVS volume, expressed in cubic centimeters (cm^3^) (Figure 1).

The reproducibility of this method has been demonstrated to be excellent, with intraclass correlation coefficients (ICC) reaching 0.97 for single measurements and 0.98–0.99 for average measurements [20].

### 2.2. Statistical Analysis

Distribution normality was assessed using the Shapiro-Wilk test. Normally distributed variables are presented as mean ± standard deviation (SD), whereas non-normally distributed variables are reported as median and interquartile range (IQR). Given the non-normal distribution observed for several variables, both parametric and non-parametric analyses were performed as appropriate. Between-group comparisons were performed using the Mann–Whitney U test for two-group comparisons and the Kruskal–Wallis test for comparisons involving three or more groups. Effect sizes were estimated using Cohen’s d. Associations between IVS volume and continuous variables were assessed using Pearson’s or Spearman’s correlation coefficients, according to data distribution. Multivariable analysis was performed using Ordinary Least Squares (OLS) linear regression (IVS_Volume = β_0_ + β_1_ × Gestational_Age + β_2_ × Maternal_Obesity) to evaluate the independent association between maternal obesity and IVS volume after adjustment for gestational age. Multicollinearity was assessed using the variance inflation factor (VIF). Analysis of covariance (ANCOVA) was additionally used to estimate adjusted group means corrected for covariates. Residual diagnostics included assessment of normality using the Shapiro–Wilk test and evaluation of homoscedasticity. Model goodness-of-fit was assessed using the coefficient of determination (R^2^). This study was conducted in accordance with the principles of the Declaration of Helsinki. All data were anonymized prior to analysis and handled in compliance with the General Data Protection Regulation (GDPR). Institutional Review Board approval was obtained. Given the retrospective observational design and the use of fully de-identified clinical data extracted from electronic medical records, no prospective planning or informed consent was required per Italian and European regulations (D.Lgs 196/2003, GDPR Art 89). Differences were considered statistically significant with *p* < 0.05. Data were analyzed using Python (version 3.12) with SciPy and Statsmodels libraries. Ethical clearance number: CET Marche Prot. No 2022-117, 9 June 2022.

## 3. Results

A total of 88 consecutive pregnancies were enrolled, all of which underwent volumetric assessment of the fetal heart using the STIC-VOCAL technique. The study was exploratory and included all eligible consecutive cases during the study period; therefore, no a priori sample size calculation was performed. The study included 88 pregnancies, with a mean maternal age of 31.2 ± 5.5 years (range, 17–41 years). Participants were equally distributed between the non-obese group (n = 44, 50.0%) and the obese group (n = 44, 50.0%). The demographic and clinical characteristics of the study population are summarized in Table 1.

Correlation analyses between IVS volume and continuous variables are summarized in Table 2.

Strong and highly significant positive correlations were observed between IVS volume and both gestational age (Pearson’s r = 0.891, *p* < 0.0001; Spearman’s ρ = 0.920, *p* < 0.0001) as well as estimated fetal weight (Pearson’s r = 0.860, *p* < 0.0001; Spearman’s ρ = 0.927, *p* < 0.0001). In contrast, IVS volume was not significantly associated with maternal BMI, either pre-pregnancy (r = 0.059, *p* = 0.587) or current (r = 0.088, *p* = 0.414), nor with maternal age (r = 0.041, *p* = 0.707).

**Figure 2:**
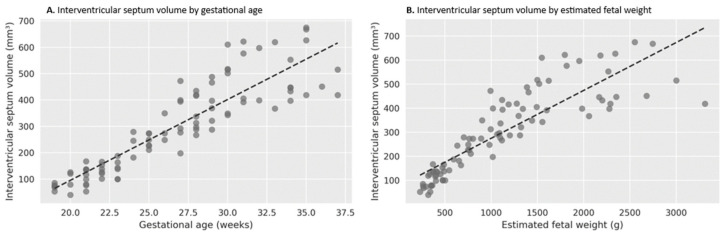
Scatterplots showing the relationship of IVS volume with gestational age (**A**) and estimated fetal weight (**B**).

As shown in Figure 2, IVS volume progressively increased throughout gestation and with increasing fetal growth, demonstrating a nearly linear relationship across the analyzed range.

As depicted in Table 3, univariate analysis revealed no significant differences in IVS volume across maternal BMI categories (Kruskal–Wallis H = 1.009, *p* = 0.604). Mean IVS volume was 259.5 ± 135.3 mm^3^ in normal-weight women (BMI 18.5–24.9 kg/m^2^), 312.9 ± 133.3 mm^3^ in overweight women (BMI 25–29.9 kg/m^2^), and 311.2 ± 204.0 mm^3^ in obese women (BMI > 30.0 kg/m^2^).

Similarly, comparison between obese and non-obese groups showed no statistically significant difference in IVS volume (Mann–Whitney U = 987.5, *p* = 0.874; Cohen’s d = 0.131).

Table 4 shows the multiple linear regression analysis adjusted for gestational age, revealing a different pattern of association. In the multivariable model (IVS_Volume = β_0_ + β_1_ × Gestational_Age + β_2_ × Maternal_Obesity), both gestational age and maternal obesity emerged as significant independent predictors of IVS volume.

**Figure 3:**
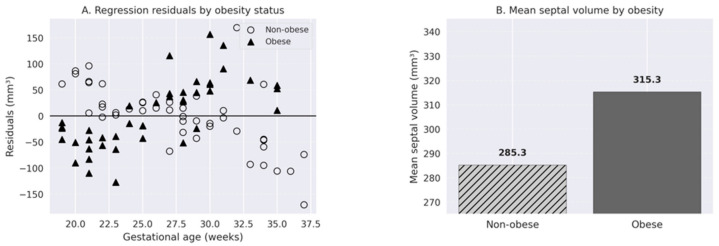
Effect of maternal obesity on IVS volume after adjustment for gestational age. Panel (**A**) shows regression residuals stratified by obesity category. Panel (**B**) shows adjusted mean IVS volumes in obese and non-obese women.

Gestational age was strongly associated with IVS volume, with an estimated increase of 32.5 mm^3^ per additional week of gestation (*p* < 0.0001). After adjustment for gestational age, maternal obesity remained independently associated with increased IVS volume (β = 89.1 mm^3^, *p* < 0.0001; 95% CI, 60.7–117.5). The multivariable model explained 85.8% of the variance in IVS volume (R^2^ = 0.858; adjusted R^2^ = 0.855). Residual diagnostics confirmed normality and homoscedasticity across BMI groups (Figure 3A). Adjusted mean IVS volumes according to obesity status are shown in Figure 3B (Figure 3B).

To further evaluate whether the relationship between gestational age and IVS volume differed according to maternal obesity status, an interaction model including gestational age, obesity status, and their interaction term was constructed (R^2^ = 0.93). In non-obese women, IVS volume increased by approximately 24.5 mm^3^ per week of gestation, whereas in obese women, the increase reached approximately 42.5 mm^3^ per week.

**Figure 4:**
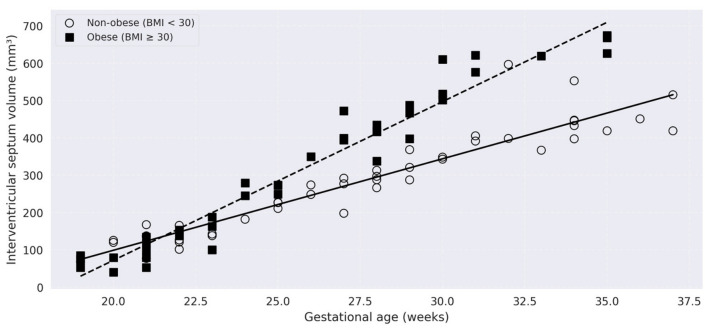
Relationship between gestational age and interventricular septum volume in non-obese (open circles, solid line) and obese women (black squares, dashed line).

The interaction between gestational age and obesity status was positive and highly significant (β = 18.0 mm^3^/week, *p* = 3.7 × 10^−13^), indicating a significantly steeper increase in IVS volume across gestation in obese compared with non-obese women (Figure 4).

## 4. Discussion

To our knowledge, this is among the first studies to evaluate the independent association between maternal obesity and fetal IVS volume using STIC-VOCAL volumetry. Notably, this relationship was not apparent on univariate analysis and emerged only after adjustment for gestational age in multivariable regression models. This finding is relevant, as gestational age is the main determinant of absolute fetal cardiac volumes and may therefore mask more subtle associations with maternal comorbidities when not adequately accounted for. By controlling for gestational age, multivariable regression allowed the specific contribution of maternal BMI to be isolated, revealing that fetuses from obese pregnancies had slightly but significantly greater IVS volumes than those from non-obese pregnancies at comparable gestational ages. Although modest in absolute terms, the observed increase in IVS volume may be biologically meaningful, and several mechanisms may underlie the association between maternal obesity and fetal septal enlargement. Pregnancies complicated by maternal obesity are characterized by an adverse intrauterine milieu, including impaired placental function and increased inflammatory signaling, and disturbances in maternal–fetal nutrient transfer [21]. These hemodynamic alterations may affect fetal cardiac loading conditions and contribute to compensatory myocardial remodeling [13,15]. In parallel, maternal obesity is frequently associated with insulin resistance, which may promote fetal hyperinsulinemia [22]. As insulin is an important fetal growth factor [15], increased fetal insulin exposure may stimulate myocardial growth, including growth of the IVS [23]. Additionally, metabolic and inflammatory pathways may also be involved. Elevated maternal free fatty acids, a typical characteristic of obesity, can cross the placenta and can trigger inflammatory and lipotoxic processes within fetal tissues, potentially contributing to structural cardiac remodeling [24,25,26]. Moreover, obesity-related placental dysfunction may impair fetal oxygenation, leading to relative hypoxia and activation of adaptive cardiac growth pathways [5,27]. Taken together, these mechanisms suggest that increased IVS volume may reflect an early structural response of the fetal heart to the metabolic, inflammatory, and hemodynamic disturbances associated with maternal obesity. However, data specifically addressing quantitative fetal cardiac volumetry by three-dimensional ultrasound in relation to maternal BMI remain limited. Existing studies have established the feasibility and reference ranges of fetal IVS volumetry using STIC-VOCAL and have applied this approach mainly in pregnancies complicated by diabetes rather than isolated maternal obesity [10,24,28]. In this context, our findings provide novel evidence that maternal obesity may be associated not only with functional cardiac changes, but also with measurable alterations in fetal cardiac structure.

### Study Limitations

Several limitations should be acknowledged. First, the sample size of 88 participants was relatively small. Although the study may have been adequately powered to detect moderate-to-large effects, it may have been underpowered to identify more subtle associations or higher-order interactions. Second, the single-center design may limit the generalizability of the findings to populations with different ethnic, genetic, or clinical characteristics. Third, the absence of postnatal follow-up limits our ability to relate antenatal IVS volume measurements to neonatal outcomes or to functional cardiac correlates in early life. Additional limitations relate to ultrasound acquisition and volumetric analysis. VOCAL-based assessment is semi-automated and operator-dependent; although all examinations were performed by experienced sonographers, inter-observer reproducibility was not directly assessed, leaving some uncertainty regarding measurement robustness. Finally, maternal metabolic factors were not systematically evaluated. The lack of data on glucose levels, lipid profile, adipokines, and other markers of metabolic dysfunction precluded a more refined assessment of their potential contribution to fetal cardiac volume.

## 5. Conclusions

This study provides evidence of an independent association between maternal obesity and increased IVS volume after adjustment for gestational age. This finding suggests that maternal obesity may be associated with early structural remodeling of the fetal heart, beyond the expected effects of fetal growth. Although the absolute magnitude of the observed difference was modest, the detection of altered IVS volume by means of STIC-VOCAL volumetry may provide a sensitive marker of fetal cardiac adaptation to an adverse maternal metabolic environment. Moreover, maternal BMI should be considered in the development of future echocardiographic reference curves for fetal cardiac volumetric parameters and screening for postnatal cardiovascular health. Further research in larger cohorts, with postnatal follow-up and incorporation of maternal and fetal metabolic biomarkers, is needed to clarify the mechanisms and clinical significance of these structural alterations.

## Figures and Tables

**Table 1:** Demographic and clinical characteristics of the cohort (*n* = 88).

Parameter	Mean ± SD	Range
Maternal age (years)	31.2 ± 5.5	17–41
Pre-pregnancy BMI	29.9 ± 8.6	18–45
Current BMI	31.7 ± 7.9	19–46
BMI increment (kg/m^2^)	1.8 ± 1.7	−1.0–9.0
Parity	0.56 ± 0.93	0–6
Gestational age (weeks)	26.7 ± 5.0	19–37
Estimated fetal weight (g)	1126 ± 745	231–3311
IVS volume (mm^3^)	299.9 ± 172.5	40.0–674.5

**Table 2:** Correlation analyses of IVS volume with continuous maternal and fetal characteristics.

Variable	Pearson’s r	Value	Spearman’s ρ	*p* Value
Maternal age	0.0407	0.7068	0.0727	0.5011
Pre-pregnancy BMI	0.0587	0.5868	0.0091	0.9328
Current BMI	0.0882	0.4141	0.0464	0.6680
BMI increment	0.1117	0.3002	0.1774	0.0982
Gestational age	0.8910	<0.0001	0.9198	<0.0001
Estimated fetal weight	0.8596	<0.0001	0.9270	<0.0001

**Table 3:** Distribution of IVS volume according to maternal BMI category. Values are presented as mean ± SD and median (range) for descriptive purposes. (Kruskal–Wallis test: *p* = 0.604). NS, not significant.

BMI Category	n	Mean (mm^3^)	SD	Median	Range	Significance
Normal weight	20	259.5	135.3	252.3	77–516	Ref
Overweight	24	312.9	133.3	317.0	68–597	NS
Obese	44	311.2	204.0	276.3	40–674	NS

**Table 4:** Multivariable linear regression evaluating predictors of IVS volume. (R^2^ = 0.858; adjusted R^2^ = 0.855; F = 257.7; *p* < 0.0001).

Predictor	β Coefficient	SE	t Statistic	*p* Value	95% CI
Intercept	−611.7	41.0	−14.93	<0.0001	[−693.2, −530.3]
Gestational Age	32.50	1.44	22.64	<0.0001	[29.6, 35.4]
Maternal Obesity	89.10	14.3	6.23	<0.0001	[60.7, 117.5]

SE, standard error; CI, confidence interval.

## Data Availability

The data presented in this study are available on request from the corresponding author. (the data are not publicly available due to privacy or ethical restrictions.)
